# Non-alcoholic Fatty Liver Disease and Alcohol-Related Liver Disease: Two Intertwined Entities

**DOI:** 10.3389/fmed.2020.00448

**Published:** 2020-08-20

**Authors:** Francisco Idalsoaga, Anand V. Kulkarni, Omar Y. Mousa, Marco Arrese, Juan Pablo Arab

**Affiliations:** ^1^Departamento de Gastroenterología, Escuela de Medicina, Pontificia Universidad Católica de Chile, Santiago, Chile; ^2^Department of Hepatology, Asian Institute of Gastroenterology, Hyderabad, India; ^3^Division of Gastroenterology and Hepatology, Mayo Clinic, Rochester, MN, United States; ^4^Division of Gastroenterology and Hepatology, Mayo Clinic Health System, Mankato, MN, United States; ^5^Centro de Envejecimiento y Regeneración (CARE), Facultad de Ciencias Biológicas, Pontificia Universidad Católica de Chile, Santiago, Chile

**Keywords:** non-alcoholic fatty liver disease, steatosis, cirrhosis, NAFLD, NASH, alcohol, alcohol-related liver disease, ALD

## Abstract

Non-alcoholic fatty liver disease (NAFLD) is the most common cause of chronic liver disease worldwide, with a prevalence of 25–30%. Since its first description in 1980, NAFLD has been conceived as a different entity from alcohol-related fatty liver disease (ALD), despite that, both diseases have an overlap in the pathophysiology, share genetic–epigenetic factors, and frequently coexist. Both entities are characterized by a broad spectrum of histological features ranging from isolated steatosis to steatohepatitis and cirrhosis. Distinction between NAFLD and ALD is based on the amount of consumed alcohol, which has been arbitrarily established. In this context, a proposal of positive criteria for NAFLD diagnosis not considering exclusion of alcohol consumption as a prerequisite criterion for diagnosis had emerged, recognizing the possibility of a dual etiology of fatty liver in some individuals. The impact of moderate alcohol use on the severity of NAFLD is ill-defined. Some studies suggest protective effects in moderate doses, but current evidence shows that there is no safe threshold for alcohol consumption for NAFLD. In fact, given the synergistic effect between alcohol consumption, obesity, and metabolic dysfunction, it is likely that alcohol use serves as a significant risk factor for the progression of liver disease in NAFLD and metabolic syndrome. This also affects the incidence of hepatocellular carcinoma. In this review, we summarize the overlapping pathophysiology of NAFLD and ALD, the current data on alcohol consumption in patients with NAFLD, and the effects of metabolic dysfunction and overweight in ALD.

## Introduction

Non-alcoholic fatty liver disease (NAFLD) and alcohol-related liver disease (ALD) are the most frequent causes of chronic liver disease worldwide (1, 2). Over the past decade, both entities have been increasing in the U.S. and worldwide, contributing to the rising burden of cirrhosis and hepatocellular carcinoma (HCC) and surpassing the figures of viral hepatitis infection as chief etiologies of these conditions (3). These temporal trend shifts in the contributions of NAFLD and ALD to the total burden of liver disease are likely related to diverse factors. Among them are the changing epidemiology of viral hepatitis in the last decade, the increasing rates of obesity and type 2 diabetes (T2DM) and the changing patterns of alcohol consumption in the general population. NAFLD and ALD have a number of commonalities and may eventually coexist in the same individual. In this context, it seems timely to review some basic and clinical concepts on these two intertwined conditions.

NAFLD is closely related to obesity and overweight as well as to the presence of metabolic dysfunction, and although the occurrence of steatosis in this setting was recognized in the early 1950s (4), only in 1980 was it pointed out as a possible cause of cirrhosis in a landmark case series study by Ludwig et al. (5). At the present time, NAFLD is defined by steatosis associated with a spectrum of hepatic histopathologic changes including the presence of inflammatory infiltrates and various degrees of fibrosis and cirrhosis (1). These features develop in the absence of known factors that cause fat accumulation such as alcohol consumption (defined as <30 g/day in men and <20 g/day in women), viral liver disease, and hereditary disorders. NAFLD is usually found in patients with comorbidities, such as metabolic syndrome (MetS), obesity, insulin resistance (IR), T2DM, and dyslipidemia (6). It is estimated that between 7 and 30% of patients with NAFLD may develop an inflammatory subtype termed non-alcoholic steatohepatitis (NASH), which is hallmarked by the presence of cell ballooning and lobular inflammation (7). NASH seems to be a more aggressive form of the disease that progresses more commonly to advanced fibrosis and cirrhosis (8). Patients with NAFLD, particularly those with NASH, have an increased mortality due to liver disease, and it is likely that cardiovascular mortality could also be increased (9).

NAFLD has increased significantly worldwide over the last decades, in line with the obesity epidemic and sedentary lifestyles (8, 10–12). Currently, the global prevalence of NAFLD is around 25%, with important differences between the Middle East (32%), South America (31%), United States (24.1%), and Africa (14%) (12–15). Additionally, the prevalence varies in association with metabolic diseases. NAFLD can be detected with ever greater prevalence in ~90% of obese patients and 65% of overweight patients (13) and in up to 70% of T2DM patients (16).

ALD affects 2–2.5% of the general population and exhibits a greater prevalence in areas with higher alcohol consumption (17). In Western countries, up to 50% of the patients with end-stage liver disease have alcohol as a major etiologic factor (18). According to the World Health Organization in 2018, more than 3 million deaths every year—representing around 5% of global deaths—are attributable to alcohol consumption (19). In the United States in 2006, alcohol-related deaths (excluding accidents) accounted for 22,073 deaths, with 13,000 of those specifically attributed to ALD (20). ALD is caused by heavy chronic alcohol consumption. Heavy or hazardous drinking is defined as consumption of more than 3 standard drinks per day in men, and more than 2 drinks per day in women, or binge drinking (defined as more than 5 standard drinks in men and more than 4 in women over a 2-h period) (21), implying a greater risk of developing health problems associated with alcohol (22–24). Clinical manifestations range from no symptoms to severe acute alcoholic hepatitis (AH) with or without cirrhosis (17).

The relationship between NAFLD and ALD is complex due to overlapping clinical features and lack of positive criteria for NAFLD (25). Of note, the interaction between NAFLD and alcohol consumption has been controversial over the last few years (26). Initially, some studies suggested a protective effect of moderate doses of alcohol (27). However, recent evidence indicates that there is no safe threshold for alcohol consumption in NAFLD patients (28). Moreover, alcohol use is a significant risk factor for the progression of liver disease in these individuals, eventually impacting a mortality in those patients with NAFLD and MetS (29). On the other hand, in ALD, MetS and obesity may increase liver disease progression and the incidence and mortality of HCC (30).

In this review, we aim to summarize current data on the overlapping pathophysiology of NAFLD and ALD as well as the available information on alcohol consumption in patients with NAFLD and the effects of MetS and overweight in ALD. We underscore the need for a change in NAFLD nomenclature in order to account for the dual etiology of liver disease, which is present in a likely significant proportion of patients with concurrent alcohol consumption and metabolic disturbances. Proper consideration of these concepts should impact clinical management.

## How Much Alcohol Is Bad in NAFLD?

Definition of NAFLD, and the distinction from ALD, is based on the amount of alcohol consumed, which has been established arbitrarily, and without definitive evidence. The threshold of alcohol consumption that rules out NAFLD usually is 20 g (2 units per day) in women, 30 g (3 units per day) in men, based on guidelines of scientific associations recommendations (1, 6). Alcohol consumption is reported in up to two-thirds of patients with NAFLD in the United States (31). The effect of alcohol consumption on the prognosis of NASH has been a subject of controversy for many years, with some studies suggesting a protective effect and others suggesting an increased risk of liver disease progression and HCC (32–35) (Table 1). Initial evidence for the protective effect of moderate alcohol intake in NASH dates back to 2001. Dixon et al. suggested that moderate alcohol consumption reduces the risk of NAFLD in the severely obese, probably by reducing IR (OR, 0.35; 95% CI, 0.12–1.00). Steatosis was diagnosed by laparoscopic biopsies during bariatric surgery and NASH was present only in 25% (26/105) of the cases (36). In 2007, Suzuki et al. performed a cross-sectional study in men without chronic liver diseases to determine the association between alcohol consumption (none, light, moderate, and excessive) and elevated serum aminotransferase levels. They concluded that excessive alcohol consumption was associated with increased aminotransferase levels, while light and moderate alcohol intake may protect against the development of elevated aminotransferases (37). However, aminotransferases are poor screening tools for NAFLD. The third National Health and Nutrition Examination Survey (NHANES) compared patients with no alcohol intake (*n* = 7,211) vs. patients who drank wine exclusively (up to 10 g/day) (*n* = 945). In this study, the low-dose wine consumption (but not beer or liquor) was associated with a decreased risk of elevated aminotransferase levels (OR 0.62; 95% CI 0.41–0.92) (56). In 2009, Gunji et al. developed a cross-sectional study in the Japanese population, including a large series of asymptomatic male subjects. Alcohol intake was defined through a questionnaire, and steatosis status was assessed by aminotransferases or ultrasonography (US) (38). They reported an inverse association between alcohol consumption and steatosis, with a protective effect of light and moderate alcohol intake. Later, the same group obtained similar results defining steatosis through a CT scan (independent of MetS or physical activity) (39). Another cross-sectional study in males on regular health check-ups was conducted by Hiramine et al. They utilized a questionnaire to determine the alcohol intake and classified the subjects according to alcohol consumption as none, light, moderate, and heavy drinkers (0, <20, 20–59, and ≥60 g/day, respectively). Steatosis was defined by US. They also concluded that alcohol consumption plays a protective role against fatty liver in men. It is interesting that the analysis of the drinking patterns revealed that the prevalence of fatty liver was inversely associated with the frequency of alcohol consumption (≥21 days/month) (OR 0.62, CI 0.53–0.71), but not with the volume of alcohol consumed (40). Moriya et al. reported a significant inverse correlation between drinking frequency and the prevalence of fatty liver (*p* < 0.001) in the Japanese population. These authors described that drinking <20 g on 1–3 days/week was associated with a lower prevalence of fatty liver assessed by US (adjusted odds ratio, 0.47; 95% confidence interval, 0.23–0.96). This study included men and women, obtaining the same results for both (men: OR 0.59; 95% CI 0.52–0.68; women: OR 0.60; 95% CI 0.45–0.80) (41). These results were consistent with the study by Hamaguchi et al., who defined the prevalence of steatosis by CT scan (42).

**Table 1:** Studies assessing the effect of moderate alcohol intake on NAFLD.

**References**	**Description**	**Findings**
Dixon et al. (36)	105 patients who underwent bariatric surgery (cross-sectional cohort study). Alcohol intake was studied by questionnaire and liver disease by biopsy	Moderate alcohol consumption was associated with a decreased incidence of NASH (OR, 0.35; 95% CI, 0.12–1.00)
Suzuki et al. (37)	1,177 male subjects, 5 years of follow-up (cross-sectional and prospective study). Alcohol intake assessment by questionnaire and liver disease by elevation of ALT	Alcohol consumption was negatively associated with elevated ALT (HR 0.4; 95% CI 0.1–0.9)
Dunn et al. (32)	7,211 subjects none alcohol intake and 945 wine drinkers (cross-sectional study). Alcohol consumption assessment by questionnaire and liver disease by raised ALT	Mild wine consumption was associated with 50% reduced risk of elevated ALT (OR 0.51; 95% CI 0.33–0.79) without effect in beer or liquor intake
Gunji et al. (38)	5,599 Japanese men with regular medical survey (cross-sectional study). Alcohol intake defined by Questionnaire and fatty liver detected by US	Mild (40–140 g per week) and moderate (140–280 g per week) alcohol intake reduced the risk of steatosis (OR 0.82; 95% CI 0.68–0.99 and OR 0.75; 0.61–0.93)
Gunji et al. (39)	1,138 Japanese men (≥40 years) (cross-sectional study). Alcohol intake assessment by questionnaires and fatty liver by CT	Alcohol consumption was associated with a reduced risk of steatosis. This reduction was independently of MetS and physical activity
Hiramine et al. (40)	9,886 males on regular health check-ups (cross-sectional cohort). Alcohol intake studied by questionnaire and liver disease by US	Fatty liver increased with obesity and decreased with alcohol intake (light, OR 0.71, 95% CI 0.59–0.86; moderate, OR 0.55, CI 0.45–0.67; heavy, OR 0.44, CI 0.32–0.62)
Moriya et al. (41)	4,957 men and 2,155 women without liver disease (cross-sectional study). Alcohol intake assessment by questionnaire and fatty liver by US and raised ALT	The prevalence of steatosis was lower in drinkers than in non-drinkers' men and women (*p* < 0.001 for both). NAFLD was inversely associated with both amount and frequency of alcohol intake
Hamaguchi et al. (42)	8,571 Japanese men and women (cross-sectional study). Mean BMI, 22.6 kg/m^2^ alcohol consumption assessment by questionnaires and fatty liver by US	Light and moderate alcohol intake was inversely associated with fatty liver in men (OR 0.69, 95% CI 0.60–0.79 and OR 0.72, 95% CI 0.63–0.83) and women (OR 0.54, 95% CI 0.34–0.88 and OR 0.43, 95% CI 0.21–0.88)
Dunn et al. (32)	251 modest drinkers and 331 non-drinkers (cross-sectional cohort study). Alcohol intake studied by questionnaires (AUDIT test) and fatty liver by biopsy	Modest drinking reduced the odds of NASH (OR 0.56, 95% CI 0.39–0.84), fibrosis (OR 0.56; 95% CI 0.41–0.77) and ballooning (OR 0.66, 95% CI 0.48–0.92) vs. lifetime non-drinking habits
Hagstrom et al. (43)	120 subjects with demonstrated NAFLD by biopsy (cross-sectional, cohort study). Alcohol intake assessment by questionnaires for lifetime alcohol intake and phosphatidylethanol (PEth) for recent alcohol consumption	Alcohol intake (up to 13 U per week) was associated with reduced risk of fibrosis (OR 0.86 95% CI 0.76–0.97), but high PEth was associated with increased risk of fibrosis (OR 2.77, 95% CI 1.01–7.59)
Kwon et al. (33)	77 subjects with NAFLD demonstrated by biopsy (cross-sectional cohort study). Alcohol intake assessment by retrospective questionnaire and liver disease by biopsy	Lifetime alcohol intake ≥24 g-years was associated with less severe disease (OR 0.26, 95% CI 0.07–0.97)
Moriya et al. (44)	3,773 men and 1,524 women (prospective analysis). Alcohol consumption defined by questionnaire and fatty liver by US	Modest alcohol intake was associated lower incidence of steatosis in men and woman. In men, steatosis was also reduced by alcohol intake in the range ≥280 g per week, after adjustment for confounders (OR 0.68; 95% CI 0.58–0.79)
Mitchell et al. (45)	187 NAFLD patients (cross-sectional, cohort study). Questionnaires for previous and actual alcohol intake and Liver biopsy	Mild alcohol consumption was associated with a decreased risk of advanced fibrosis (OR 0.33, 95% CI 0.14–0.78). Wine intake (not beer drinking) was negatively associated with advanced fibrosis (OR 0.20, 95% CI 0.06–0.69), compared with patients without alcohol intake
Hajifathalian et al. (27)	4,568 subjects follow-up of 70 months (prospective study). Questionnaire for amount and type of alcohol drinking and Hepatic Steatosis Index for determinate the liver disease	Mild alcohol (0.5–1.5 U per day) consumption was associated with decreased overall mortality (HR 0.64, 95% CI 0.42–0.97). However, in NAFLD alcohol consumption ≥1.5 U per day had a harmful effect on mortality (HR 1.45, 95% CI 1.01–2.10)
Sookoian et al. (46)	Meta-analysis. Included 43,175 individuals of 8 studies with high heterogeneity	Modest alcohol consumption was associated with a protective effect in NASH (fixed models: OR 0.69, 95% CI 0.65–0.73; random models: OR 0.68; 95% CI 0.58–0.81)
Ajmera et al. (26)	Critical review of 7 observational studies	Concluded a positive association between moderate alcohol use and decreased NASH and fibrosis. Heavy episodic drinking may accelerate fibrosis progression and moderate alcohol intake may increase the risk of HCC (in patients with advanced fibrosis)
Becker et al. (23)	13,285 men and women (prospective cohort study). Follow-up of 12 years. The alcohol intake assessment by a self-administered questionnaire. Alcohol-induced liver disease by death certificates/hospital registers	Alcohol intake associated with lower risk of liver disease (up to 1–6 U per week). The relative risk was significantly >1 at 7–13 U per week for women and 14–27 for men
Bellentani et al. (47)	6,917 subjects of the general population (community-based study). The alcohol intake assessment by questionnaire and liver disease by blood test and clinical	Increased risk of chronic liver disease and cirrhosis with alcohol intake above 30 g/day
Bellentani et al. (48)	257 participants (Dionysos Study, Cross-sectional, Cohort). Validated questionnaire for alcohol intake and NAFLD by US	Fatty liver risk 2.8-fold higher in drinkers (95% CI, 1.4–7.1) and 4.6-fold higher in obese persons (95% CI, 2.5–11.0). In subjects obese and alcohol intake was 5.8-fold higher (95% CI, 3.2–12.3)
Bedogni et al. (49)	144 subjects without and 336 with fatty liver (cohort study). Follow-up of 8.5 years. Questionnaire for amount of alcohol intake	The alcohol intake increases the incident steatosis by 17%, steatosis remission decreased by 10%, and mortality increased by 10%
Eckstedt et al. (34)	71 patients with NAFLD by biopsy (cohort study). Follow-up of 13.8 years. Alcohol intake assessment by validated questionnaire and oral interview. The outcome was fibrosis progression by biopsy	Episodic and continuous heavy drinking was more common among those with fibrosis progression. Binge drinking predicted fibrosis progression
Aberg et al. (30)	6,732 subjects without liver disease, follow-up of 11.4 years (cohort study). Alcohol intake studied by questionnaire and outcome was liver disease progression, HCC, liver-related death	Alcohol intake (below the risk threshold) remained as a significant independent predictor of liver disease progression and HCC
Chang et al. (50)	58,927 Korean adults with NAFLD and low fibrosis (cohort study). Followed for a median of 4.9 years. Fibrosis was assessed using non-invasive indices including NAFLD fibrosis score (NFS) and Fibrosis-4 Index (FIB-4)	Non-heavy alcohol consumption, especially moderate alcohol consumption, was significantly and independently associated with worsening of non-invasive markers of fibrosis
Younossi et al. (29)	4,264 individuals with hepatic steatosis (retrospective cohort study). Mean age, 45.9 years; 51% male; 76% white; 46% with MetS; 6.2% with excessive alcohol use. Steatosis determined by US and alcohol intake by questionnaire	The presence of MetS [adjusted hazard ratio (aHR), 1.43; 95% CI, 1.12–1.83] and excessive alcohol consumption (aHR, 1.79; 95% CI, 1.21–2.66) were independently associated with an increased risk of death in individuals with hepatic steatosis; any lower average amount of alcohol consumption was not associated with mortality (all *P* > 0.60)
Ajmera et al. (51)	285 participants were modest alcohol users and 117 were abstinent (Longitudinal study). Follow-up period of 47 months. Liver was studied by biopsies and alcohol intake by questionnaire	Modest alcohol use was associated with less improvement in steatosis (adjusted odds ratio, 0.32; 95% CI, 0.11–0.92; *p* = 0.04) and level of aspartate transaminase, as well as lower odds of NASH resolution, compared with no use of alcohol
Verrill et al. (52)	100 patients with biopsy-proven alcohol-induced liver cirrhosis (retrospective study)	Abstinence from alcohol at 1 month after diagnosis of cirrhosis was the more important factor determining survival with a 7-year survival of 72% for the abstinent patients vs. 44% for the patients continuing to drink. Early drinking status is the most important factor determining long-term survival in alcohol-related cirrhosis
Sookoian et al. (53)	A Mendelian randomization study using a validated genetic variant (rs1229984 A;G) in the alcohol dehydrogenase (ADH1B) gene as a proxy of long-term alcohol exposure	The analysis of association with the disease severity showed that carriers of the A-allele had lower degree of histological steatosis (1.76 ± 0.83 vs. 2.19 ± 0.78, *P* = 0.03) and lower scores of lobular inflammation (0.54 ± 0.65 vs. 0.95 ± 0.92, *P* = 0.02) and NAFLD-Activity Score (2.9 ± 1.4 vs. 3.7 ± 1.4, *P* = 0.015) compared with non-carriers.The analysis suggests no beneficial effect of moderate alcohol consumption on NAFLD disease severity
Yi et al. (54)	504,646 Korean subjects in health maintenance visits (Cohort study). Follow-up of 10.5 years. Questionnaires for alcohol consumption, ICD-X codes for liver disease	HCC risk increases with age and alcohol consumption (for any 20 g per day)
Askgaard et al. (55)	55,917 subjects (between 50 and 64-year-old), Danish study (1993–2011). Alcohol consumption and pattern from questionnaire. Follow-up 14.9 years	Recent daily drinking associated with an increased risk of ALD cirrhosis in men (HR, 3.65; 95% CI, 2.39–5.55), compared to drinking 2–4 days per week

At least 4 cross-sectional studies suggested a protective role of alcohol consumption after defining steatosis by liver biopsy (32, 43, 45, 57). In the NIH NASH Clinical Research Network, modest alcohol consumption was associated with less steatohepatitis, hepatocellular ballooning, and fibrosis (32). In another study, alcohol intake up to 13 U/week was associated with lower fibrosis stage in NAFLD (OR 0.86 per U/week, 95% CI 0.76–0.97). Nevertheless, an elevated phosphatidylethanol (a biomarker for recent alcohol consumption) was associated with higher stages of fibrosis (43). Finally, Mitchell et al. had similar results, but particularly with wine consumption (not with beer) and non-binge pattern (45). The association of modest alcohol intake with survival in NAFLD has also been evaluated. This analysis was made using the NHANES data (1988–2010). NAFLD was diagnosed by hepatic steatosis index (HSI) in 4,568 subjects. Modest alcohol consumption was associated with a significant decrease in all-cause mortality (after a median follow-up of 70 months, and adjustment for race, physical activity, education level, T2DM, and fiber and polyunsaturated fatty acid intake) [hazard ratio (HR) 0.64, 95% CI 0.42–0.97], whereas drinking ≥1.5 drinks per day was to be associated with an increased in mortality (HR 1.45, 95% CI 1.01–2.10) (27). Currently, only one meta-analysis has been published (46), which included 43,175 individuals. It concluded that modest alcohol consumption was associated with a protective effect in NASH (fixed models: OR 0.69, 95% CI 0.65–0.73; random models: OR 0.68; 95% CI 0.58–0.81) based on 8 heterogeneous studies. The critical review by Ajmera et al. (26) of 7 observational studies concluded a positive association between moderate alcohol use and decreased NASH and fibrosis; however, heavy episodic drinking may accelerate fibrosis progression and moderate alcohol intake may increase the risk of HCC (in patients with advanced fibrosis). However, the studies had significant methodological limitations, including incomplete adjustment for confounding factors.

Despite that some studies suggest a beneficial effect of moderate alcohol consumption on the occurrence and progression of NAFLD, more recent evidence suggests that there is no safe limit for alcohol consumption and that the alcohol intake is associated with a higher risk of liver disease progression, including HCC (23, 30, 34, 36, 47–49, 54, 55, 58–63). The association between alcohol intake and liver damage has been reported widely since 1957 (64). Many studies showed that alcohol consumption is associated with increased prevalence and progression of NASH. In the Dionysos Study, 144 subjects without steatosis and 336 with steatosis were followed up for 8.5 years. The most relevant risk factor for steatosis incidence and remission, as well as a predictor of mortality in these patients with fatty liver, was alcohol intake (20 g/day). The incidence of fatty liver increased by 17%, steatosis remission decreased by 10%, and mortality increased by 10% in the fatty liver cohort (49). Follow-up liver biopsy in 71 patients with NAFLD showed that fibrosis progression was associated with episodic drinking (at least once per month) and higher weekly alcohol consumption. Also, the heavy episodic drinking (*p* < 0.001) and IR (*p* < 0.01) were independently associated with significant fibrosis progression. The study concluded that moderate alcohol consumption was associated with fibrosis progression in NAFLD, and the authors advised to refrain from heavy episodic drinking in patients with NAFLD (34). Recent studies showed that there is no safe limit for alcohol consumption and suggested that even light alcohol consumption is not safe in NAFLD. In 2018, a systematic analysis from the Global Burden of Diseases, Injuries, and Risk Factors Study, which included 28 million individuals and 649,000 cases with outcomes, also suggested a detrimental effect of moderate alcohol intake (65). A Finnish cohort study of 6,732 individuals without baseline liver disease, after a follow-up of 11.4 years, demonstrated that non-risky alcohol intake (<3 units per day in men and 2 units per day in women) was associated with a significant increase in the risk of liver disease progression (30). A large-scale cohort was performed in 58,927 young and middle-aged Korean individuals with NAFLD (with low baseline fibrosis scores), who were followed for a median of 4.9 years. The progression from low to intermediate or high probability of advanced fibrosis was assessed using non-invasive index including NAFLD fibrosis score (NFS) and Fibrosis-4 Index (FIB-4). They demonstrated that light (1.0–9.9 g/d) or moderate (10.0–29.9 g/d for men and 10.0–19.9 g/d for women) alcohol consumption compared with none (0 g/d) was significantly and independently associated with worsening of fibrosis. The effect was higher with moderate alcohol consumption (50). The authors also suggested that a safe limit of alcohol use may not exist. In a study that analyzed NHANES III including 4,264 adults with hepatic steatosis diagnosed by US examination, the overall mortality was significantly higher among subjects with excessive alcohol intake (32.2%) vs. subjects with non-excessive alcohol consumption (22.2%) after 5 and 20 years of follow-up (*p* = 0.003). The association of excessive alcohol use with mortality was significant in individuals who have MetS (aHR, 2.46; 95% CI, 1.40–4.32) but not without it (*p* = 0.74) (29). The impact of alcohol consumption is associated not only with incidence of steatosis, fibrosis progression, and mortality but also with less improvement in steatosis in patients with NASH and increased liver malignancies. In a longitudinal analysis of liver biopsies from patients with NAFLD, the low and modest alcohol use was associated with less improvement in steatosis, higher levels of AST, and less NASH resolution, compared with no alcohol intake (51). Another study suggested that total abstinence even prevents disease progression and is the more important factor determining survival in patients with established cirrhosis (52). Additionally, data from a Mendelian randomization study using a validated genetic variant (rs1229984 A:G) in the alcohol dehydrogenase (ADH1B) gene as a proxy of long-term alcohol exposure was used, thereby minimizing measurement bias and confounding factors. They found that carriers of the A-allele consumed significantly lower amounts of alcohol compared with non-carriers. Additionally, A-allele carriers had a lower degree of histological steatosis, lobular inflammation, and NAFLD-Activity Score (NAS) compared with non-carriers. They suggested that there is no beneficial effect of moderate alcohol consumption on NAFLD disease severity (53).

The association between alcohol intake and HCC development has been evaluated. Mild or moderate alcohol consumption may be a cofactor for the development of HCC in NAFLD. In a study that included 504,646 Korean patients (age 40–80 years) on routine health checkups, HCC incidence was associated with hepatitis B and C infection, and each 20 g/day of alcohol intake increased the risk of HCC by 6, 8, 16, and 30%, respectively, in individuals aged <50, 50–59, 60–69, and 70–80 years (54). An analysis of the participants in the Health 2000 and FINNRISK (1992–2012) databases was performed, which linked national registers for hospital admissions, malignancies, and death regarding liver, cardiovascular, and malignant disease, as well as all-cause mortality. They concluded that alcohol consumption is associated with a dose-dependent risk of advanced liver disease and neoplasia but a dose-dependent decrease in cardiovascular outcomes (21% risk reduction with ≤1 unit per day intake limited to non-smokers) (58). Alcohol intake is also associated with extrahepatic cancers, particularly breast, oral, pharyngeal, and colorectal cancer (66–69).

In summary, although some studies suggest a beneficial effect of light and moderate alcohol consumption on the occurrence and progression of NAFLD, most of them are cross-sectional studies, limiting their interpretation. These studies defined the association observed on the grounds of present alcohol intake history; nevertheless, NAFLD and ALD are processes that require long-term exposure and the damage could be driven by previous alcohol history. This is limited by the recall bias. Some studies have significant methodological limitations (including incomplete adjustment for confounding factors, as metabolic history) and several potential biases (especially in retrospective analyses), limiting their validity. On the other hand, the evidence supporting no benefit or even a detrimental effect of alcohol intake is based on solid longitudinal studies. The association between alcohol intake with fibrosis progression and cancer in these studies seems appealing and less biased than observed in cross-sectional analyses.

## How Obesity and Metabolic Syndrome Affect ALD?

In recent years, the association between alcohol consumption and NAFLD progression has been clearly established. Emerging evidence has demonstrated that obesity and MetS increase the progression of ALD and HCC incidence and mortality. Indeed, a synergism between alcohol and obesity has been suggested. A cross-sectional study (48) including 257 participants of the Dionysos Study used a validated food questionnaire and ultrasound assessment of NAFLD. The prevalence of steatosis was increased in heavy drinkers (46.4% [95% CI, 34–59%]) and obese (75.8% [CI, 63–85%]) compared with controls (16.4% [CI, 8–25%]). Those heavy drinkers who are obese had an even higher prevalence of NAFLD, 94.5% (CI, 85–99%), which suggest an additive effect. Obesity doubles the risk of steatosis in heavy drinkers (48). This synergistic effect was also observed in 2 of the long-term Midspan prospective cohort studies (9,559 men) in Scotland. The body mass index (BMI) and alcohol consumption were strongly associated with liver disease mortality in analyses adjusted by other confounders (*p* = 0.001 and *p* < 0.0001, respectively). After a median follow-up of 29 years, consumers of 15 U/week or more exhibited higher rates of liver disease irrespective of BMI. In mild users (1–14 U/week), an excess of liver disease was only observed in subjects with obesity, with a synergistic effect between alcohol and BMI (synergy index, 2.89; [95% CI, 1.29–6.47]) (60). In the analysis of NHANES III, previously commented in this review, the presence of MetS [adjusted hazard ratio (aHR), 1.43; [95% CI, 1.12–1.83]] and excessive alcohol consumption (HR, 1.79; [95% CI, 1.21–2.66]) were independently associated with an increased mortality in subjects with steatosis. Additionally, alcohol intake and the presence of MetS had a synergistic effect (29). It is necessary to consider the alcohol intake as an aggravating element of overweight and obesity. A mild alcohol intake can contribute with 100–300 kcal/day, directly to weight gain and obesity, irrespective of the type of alcohol consumed (69, 70).

The association between BMI and HCC (incidence and mortality) has been demonstrated through two prospective population-based studies in Taiwan. The first of them was a prospective study that included 2,260 Taiwanese men positive for HBV infection, followed up for 14 years. HCC was diagnosed by imaging or histopathology (Cancer Registry). In this study, alcohol intake (any amount) had synergistic effects with the risk of incident HCC in analyses adjusted for age (HR, 3.41; 95% CI, 1.25–9.27; *p* < 0.025) and multiple variables (HR, 3.40; 95% CI, 1.24–9.34; *p* < 0.025). Also, the risk of HCC increased in overweight (HR, 2.4; 95% CI, 1.3–4.4), obese (HR, 2.0; 95% CI, 1.1–3.7), and extremely obese (HR, 2.9; 95% CI, 1.0–8.0) alcohol users (*p* for trend = 0.046) (62). Later, Loomba et al. conducted a prospective, population-based study of 23,712 Taiwanese, followed for 11.6 years for the incidence of HCC, and the study concluded with similar results. Alcohol consumption and obesity (BMI ≥30) showed a synergistic association with the risk of incident HCC in both unadjusted analyses (HR = 7.19, 95% CI: 3.69, 14.00; *p* < 0.01) and multivariable-adjusted analyses [age, sex, smoking, serum alanine aminotransferase (ALT), serum hepatitis B surface antigen, anti-hepatitis C virus antibody, and T2DM] (HR = 3.82, 95% CI: 1.94, 7.52; *P* < 0.01). Finally, the study concluded that obesity and alcohol have a synergic effect increasing the risk of incident HCC (63).

## New Names for NAFLD/ALD: A Changing Nomenclature for Fatty Liver Disorders

**Figure 1:**
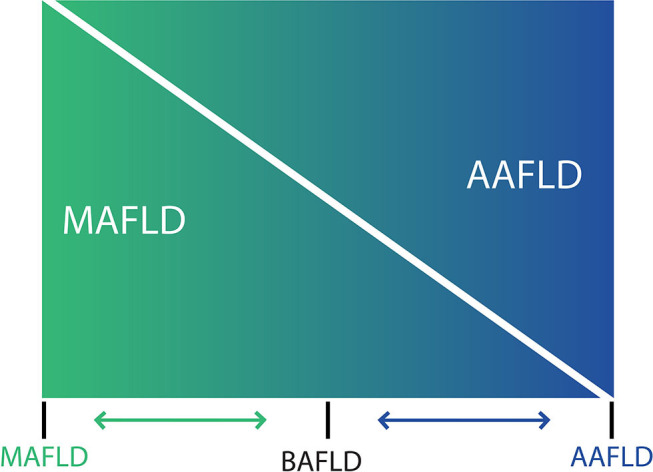
Spectrum of fatty liver diseases. In non-alcoholic fatty liver disease (NAFLD) and alcohol-related liver disease have been generally conceived as different entities, and despite that both conditions share an overlapping pathophysiology and frequently coexist in clinical practice, thresholds to diagnose NAFLD makes difficult to account for a dual etiology in a given patient. For that reason, a nomenclature change has been proposed [see (71) in the main text] considering that at the ends of the spectrum of fatty liver disease, there are patients with true ALD (now named alcohol-associated fatty liver disease, AAFLD) and some with true NAFLD with alcohol consumption near-zero (now named metabolic associated fatty liver disease, MAFLD) but that the vast majority of patients are between these two extremes. Thus, in clinical practice there will be patients with ALD that have metabolic cofactors (AAFLD with MetS) and patients with NAFLD that consume alcohol, which contributes to the disease process (MAFLD with alcohol component). In the middle, a large group of patients have both conditions (NAFLD and ALD) with some showing an equal contribution of alcohol and metabolic factors (proposedly named as both alcohol and metabolic associated fatty liver disease, BAFLD).

**Figure 2:**
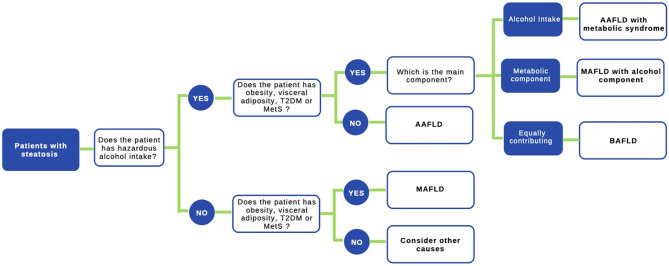
Proposed algorithm to approach patients with liver steatosis. A staggered algorithm to approach patients with liver steatosis is shown. This algorithm considers alcohol intake and metabolic cofactors (obesity, T2DM, and MetS) and classifies patients in AAFLD alcohol-associated fatty liver disease (true ALD) if hazardous alcohol intake is present, AAFLD with metabolic component (predominant ALD but with metabolic cofactors), MAFLD metabolic associated fatty liver disease (NAFLD with alcohol consumption near zero), MAFLD with alcohol component (NAFLD but with alcohol consumption contributing to the disease process), and finally BAFLD both alcohol and metabolic associated fatty liver disease (patients with both NAFLD and ALD equally contributing or no possible to determine which predominates). Criteria to diagnose MAFLD are suggested in (72) in the main text.

The use of the acronym NAFLD as an umbrella term is now recognized as a problematic issue in the field of hepatology (31). This is largely due to the significant heterogeneity of patients grouped under that denomination as well as by the absence of positive criteria for NAFLD, which makes it difficult to classify subjects with metabolic alterations drinking beyond the threshold set for NAFLD. The latter also impedes the recognition of dual etiology for liver disease in individuals with both moderate or excessive alcohol consumption and metabolic disturbances. For this reason, and in order to better characterize the disease, NAFLD nomenclature has recently been revised and a new consensus-driven acronym proposed (71). Thus, the term MAFLD, which stands for metabolic (dysfunction)-associated fatty liver disease, was suggested as a more appropriate overarching term. This revised nomenclature should allow for more precise study designs leading to decreased variability of study groups and to a better understanding of the natural history of the disease. Positive criteria to diagnose MAFLD have been also proposed by the same expert group (72), which considers the evidence of fat accumulation in the liver and presence of evidence of metabolic dysregulation. The latter is defined by the presence of at least two metabolic risk abnormalities (dyslipidemia, hypertension, abdominal obesity, prediabetes, IR, or elevated high sensitivity C-reactive protein). Since ALD also comprises a spectrum of liver lesions, some adaptations may be needed in order to acknowledge the dual etiology of patients with fatty liver disease (i.e., concomitant MAFLD and ALD). Thus, the spectrum of fatty liver disease (Figure 1) should include patients with true ALD (alcohol-associated fatty liver disease, AAFLD), patients with predominant ALD but with metabolic cofactors (ALD with MetS), those with true NAFLD with alcohol consumption near zero (MAFLD) (71), and patients with NAFLD but with alcohol consumption contributing to the disease process (i.e., MAFLD with alcohol component). A final group will be composed of those patients with both MAFLD and ALD, equally contributing (or not possible to determine which one predominates) to the disease process (both alcohol and metabolic associated fatty liver disease, BAFLD). Similarly, the term BASH has been used to describe both alcohol and metabolic associated steatohepatitis (73). Each group probably has different clinical manifestations, course, liver prognosis, and mortality. This approach recently suggested by Eslam et al. (31) proposes that patients with fatty liver and predominance of metabolic dysfunction could be stratified according to alcohol intake and patients with alcohol predominant fatty liver according to the presence of coexisting metabolic comorbidities when included in clinical studies. These distinctions may help to a more robust understanding of the natural history of these different patient populations. In Figure 2, we suggest an algorithm to be applied in patients with fatty liver, which intend to account for dual etiology and predominance in the setting of metabolic dysfunction and alcohol consumption.

## Pathogenesis of NAFLD and ALD: Overlapping Aspects and Salient Differences

**Figure 3:**
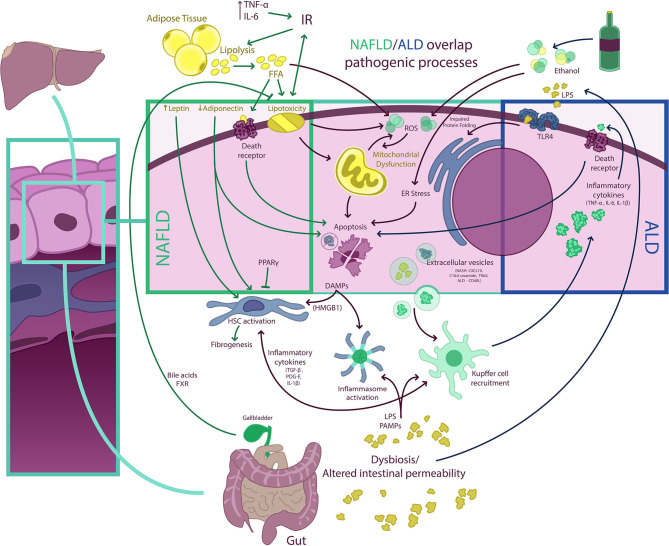
NAFLD/ALD overlapping pathogenic processes. Free fatty acids (FFA) and ethanol have a myriad of effects on hepatocytes determining, among other phenomena, the occurrence of mitochondrial dysfunction, endoplasmic reticulum (ER) stress (resulting in impaired protein folding), and excessive production of ROS, which result in hepatocellular injury, activation of other cell death pathways, and inflammasome activation. Damaged hepatocytes release damage-associated molecular pattern (DAMP) molecules [e.g., high-mobility group box 1 (HMGB1)] that signal to Kupffer and hepatic stellate cells (HSC), fueling inflammation and fibrogenesis. Extracellular vesicle (EV) release from hepatocytes also contributes to both Kupffer cell and HSC activation. In non-alcoholic fatty liver disease (NAFLD), insulin resistance (IR) is a central phenomenon promoting adipose tissue lipolysis and an increased FFA flux to the liver. This FFA overflow surpasses the storage capacity of hepatocytes and determines the occurrence of lipotoxicity and activation of cell death pathways. Also, adipose tissue dysfunction is associated with a proinflammatory state with elevated levels of circulating cytokines [e.g., tumor necrosis factor-α (TNF-α) and interleukin (IL)-6] and with an imbalance in circulating levels of adipose tissue-derived adipokines (i.e., a decrease in adiponectin and an increase in leptin). This may increase IR and contribute to HSC activation. Of note, some nuclear receptors such as peroxisome proliferator-activated receptors (PPARs) and farnesoid X receptor (FXR), a bile acid receptor, may have anti-inflammatory and anti-steatotic effects, which are being exploited therapeutically. Both NAFLD and ALD (alcohol-related liver disease) are associated with intestinal dysbiosis and altered gut permeability, which results in the pass of bacterial products [e.g., pathogen-associated molecular patterns (PAMPs), lipopolysaccharides (LPS), and others] into portal circulation. In the liver, LPS and PAMPS may activate different Toll-like receptors (TLRs) as well as promote inflammasome assembly determining amplification of inflammatory responses. In ALD, proinflammatory cytokines released from macrophages may also activate cell death receptors and induce apoptosis.

Although NAFLD and ALD are two distinct biological entities, they have a number of commonalities in their pathogenetic mechanisms leading to activation of both hepatic inflammatory and fibrogenetic pathways that fuel disease progression (74, 75) (Figure 3). Disturbed lipid handling by the hepatocyte resulting in intracellular accumulation of potentially toxic bioactive lipid species is an essential phenomenon in both NAFLD and ALD followed by the occurrence of cellular stress [i.e., endoplasmic reticulum (ER) stress and mitochondrial dysfunction] and death, which in turn triggers the innate immune response and activation of hepatic stellate cells (HSCs), resulting in inflammation and excessive collagen production and deposition (76). This general sequence is highly heterogeneous in both entities and is modulated by different genetic and epigenetic factors, some of which are also common for NAFLD and ALD. This accounts for the broad phenotypic spectrum seen in both diseases. In the following paragraphs, we summarize the main mechanisms of liver injury at play in NAFLD and ALD and underscore their similarities and differences (77–80).

### Hepatic Fat Accumulation and Lipotoxicity

Increased lipid droplets (i.e., steatosis) inside the hepatocytes is the earliest histological finding in NAFLD and ALD (78). These lipid droplets are enriched in fully saturated triglycerides and result in the typical histological pattern of macrovesicular steatosis common to both entities (7). Excessive accumulation of triglycerides and other lipid species relates to a dysregulated hepatic lipid flux consisting in an increased hepatocellular lipid uptake, synthesis, and degradation [i.e., fatty acid oxidation (FAO)], induced by a positive caloric balance and IR in NAFLD and ethanol consumption in ALD (81). The main regulators of this process are SREBP1c (82) and PPARα and PPARδ, which are critical regulators of FAO (83). In NAFLD, excessive calorie intake increases the size and number of adipocytes and renders them insulin resistant, leading to uncontrolled lipolysis and decreased fatty acid uptake, thus promoting the release of free fatty acids (FFA) into the circulation. FFA are later uptaken by hepatocytes promoting lipid droplet formation. Of note, some data suggest that fatty acid transporters are upregulated in the setting of NAFLD (84). Also, *de novo* hepatic lipogenesis (DNL) seems to be upregulated in most subjects with NAFLD, which relates to inactivation of peroxisome proliferator-activated receptor-alpha (PGC1-α) and upregulation of SREBP1c (82, 85–87). Interestingly, DNL may be accompanied by a compensatory enhancement of FAO, but studies in this regard are conflicting. Human data from patients with NAFLD show that FAO may be enhanced, unchanged or decreased (84). In ALD, ethanol consumption induces a multilevel disturbance of hepatic lipid metabolism (88). One of the best-studied effects, in murine models, is the increase in SREBP1c expression (a key transcription factor in hepatic lipogenesis) (85), leading to increased expression of hepatic lipogenic genes and increased DNL. Other important enzymes controlling lipid fluxes such as acetyl-CoA carboxylase, ACC (limiting enzyme in DNL), and carnitine palmitoyltransferase, CPT (limiting enzyme for mitochondrial β-oxidation, that regulates lipid degradation), have a pivotal role in both diseases in human studies (88, 89). Ethanol increases the activity of ACC and suppresses the rate of palmitic acid oxidation, producing modifications in fatty acid metabolism and steatosis (90). Additionally, an increased ACC expression has been described in experimental murine models of NAFLD (91). Accumulation of saturated fatty acids as well as other harmful lipids such as ceramides, diacylglycerols, and lysophosphatidylcholine, among others, promote the occurrence of lipotoxicity, a phenomenon defined by the appearance of leading to cellular dysfunction and death (92). Lipotoxicity plays an important pathophysiological role in NAFLD/NASH (93) and likely drives disease progression through different mechanisms such as direct cytotoxicity, increased IR and hyperinsulinemia, cell signaling modification [through hepatic nuclear factor-α or toll-like receptors (TLR)], ER stress, upregulation of autophagic processes, and trigger of different cell death pathways (e.g., apoptosis, necrosis, pyroptosis; see below). Cell injury and death, in murine and human models, determine release of damage-associated molecular pattern (DAMP) molecules, leading to macrophage recruitment and a secondary inflammatory response (94). In ALD, although accumulation of FFA occurs similarly to NAFLD, the lipotoxic phenomenon has not been well characterized and information on the nature of intrahepatic lipid species and their cellular effects is scarce (95).

### Insulin Resistance

IR plays a pivotal role in the pathogenesis of NAFLD. IR in adipose tissue is associated with an increase lipolysis in adipocytes, leading to excess FFA release into the circulation and to a higher uptake by the hepatocytes, which is a driver of steatosis. In turn, increased hepatic fat content promotes IR in hepatocytes by decreasing insulin-stimulated tyrosine phosphorylation of both insulin receptor substrate (IRS)-1 and IRS-2, which leads to increased gluconeogenesis and hepatic glucose production, which leads to hyperinsulinemia. The latter stimulates the transcription factor SREBP-1c, activating most genes involved in DNL, further increasing steatosis (96). Worsening IR is considered a potential driver of disease progression in NAFLD (97), and targeting IR is one of therapeutic strategies explored for NAFLD/NASH treatment according to evidence from *in vitro* and mouse models (98). With regard to the relationship between alcohol consumption and IR, data are limited. Both clinical and experimental information suggests that insulin signaling is impaired in ALD. Chronic alcohol consumption disrupts whole-body lipid metabolism, and several studies, in clinical and preclinical mouse models, suggest that alcohol may promote IR and increase the risk of T2DM (99). Indeed, it is likely that the presence of IR, which is prevalent in patients with ALD, may increase the risk of advanced liver disease through multiple mechanisms. However, current data is limited, and more studies are needed to confirm if targeting insulin signaling pathways pharmacologically may be beneficial in the setting of ALD.

### Cell Death Signaling

Another important process involved in the pathogenesis of NAFLD/ALD is the activation of cell death pathways (100–102). This pathway can be activated by both intrinsic and extrinsic signaling via surface receptors belonging to the tumor necrosis factor-alpha (TNF-α) receptor family (103–105). Specifically, TRAIL (TNF-related apoptosis-inducing ligand) receptor 2 (TRAIL-R2) has been associated with hepatocyte lipoapoptosis, probably as an effect of toxic lipids (free cholesterol, ceramides, and FFA), that promote reorganization of plasma membrane domains and ligand-independent activation of TRAIL-R2 signaling leading to cell death (106–109). In addition to apoptosis, other lytic forms of cell death may be at play in NAFLD/NASH, such as necroptosis, pyroptosis, and ferroptosis, which are related to cell-membrane permeabilization (101). The TRAIL pathway has been also implicated in ALD pathogenesis. After ethanol consumption, TRAIL expression leads to hepatic steatosis and TRAIL-mediated steatosis that can be inhibited by the neutralizing TRAIL antibody (110). Also, ER stress, induced by alcohol consumption and lipotoxicity, and reactive oxygen species may activate Bcl2 initiators of apoptosis members (108) and inhibit guardian members, inducing cell deaths, through caspase activation in mouse models (111). Finally, activation of apoptosis signal-regulating kinase 1 (ASK-1), which leads to phosphorylation of p38 and JNK and activation of several stress response pathways, has been involved in apoptosis occurrence in both NASH and alcoholic hepatitis. This is of particular interest since inhibitors of this pathway are available (i.e., selonsertib). However, studies conducted to date have yielded negative results (112).

Other cell death pathways that have been studied in liver diseases is necroptosis, which is a form of programmed necrosis. Necroptosis is defined as a non-apoptotic cell-death-receptor-mediated death observed in some cell types and is dependent of a kinase cascade that involves a family of proteins known as receptor interaction protein kinases (RIPKs), in particular RIPK3 activation of mixed-lineage-kinase-domain-like (MLKL) (113). This pathway has been explored in mouse models of NAFLD and ALD with different results (113–116). Of note, RIPK3-dependent and RIPK1-independent activity activation has been described in ALD, while in NASH models studies are contradictory (116–118). Currently, new studies assessing the role of necroptosis in fatty liver are underway (119).

Autophagy, or cellular self-digestion, is a cellular pathway crucial for development, differentiation, homeostasis, and survival of cells. This process is used to eliminate potentially harmful proteins and organelles and to remove intracellular microbial pathogens. In ALD and NAFLD, a dysregulation of this process with a decreased autophagic function that can lead to liver cell death, steatohepatitis, and HCC exist. In the setting of NASH, it has been demonstrated that palmitic acid suppresses autophagy, while oleic acid may promote it. Also, mice with genetic deletion of *Atg7* (a critical autophagy mediator) have shown increased hepatic fat content accumulation (120). The autophagic degradation of intracellular lipid droplets may play a role in buffering FFA toxicity and maintaining hepatic lipid homeostasis (120–123). In ALD, a normal functioning of autophagy is associated with attenuated alcohol-induced injury and less lipotoxicity (122).

### Immune Response

Innate immune cells have a fundamental role in the pathogenesis of both NAFLD and ALD, sharing important characteristics but with some substantial differences (124, 125). Release of DAMPs from hepatocytes activate innate immune cells, particularly resident macrophages (i.e., Kupffer cells) (123) in *in vitro* studies using cultured HepG2 cells and primary mouse hepatocytes. Additionally, some specific DAMPs such as high-mobility-group protein box 1 (HMGB1) have shown to activate TLR4 in NASH and ASH, playing a pivotal role during the early progression of NAFLD (126, 127). Other DAMPs, for example, sonic hedgehog (SHH) ligands, have been also associated with progression of NAFLD and fibrosis in human studies (128).

Neutrophils are another key element in ASH and NASH. Neutrophil elastase (NE), a protease released by neutrophils, produces cellular IR, and the deletion of NE produces less tissue inflammation and is associated, in mouse models, with lower adipose tissue neutrophil and macrophage content (129). In ALD, neutrophils induce progression through the release of ROS, proteases, and proinflammatory mediators (130). Additionally, neutrophils have been associated with portal hypertension as these immune cells promote the formation of microvascular thrombosis, through neutrophil extracellular traps (NETs). Occurrence of microvascular thrombosis and fibrin may drive portal hypertension through space effects in liver sinusoids (131).

Finally, the monocyte chemoattractant protein-1 (132) is another important component in ASH and NASH. The monocyte chemotactic protein-1 (MCP-1) is an inflammatory chemokine released by hepatocytes, Kupffer cells, sinusoidal endothelial cells, and hepatic stellate cells in response to alcohol, producing chemoattraction of macrophages and maintaining mononuclear infiltration in mouse models (133–136).

### Inflammasome

Inflammasomes are multiprotein complexes that are mainly expressed in hepatocytes and myeloid cells (i.e., Kupffer cells) (137) that sense pro-inflammatory signals through NOD-like receptors (NLRs) and activate caspase-1, the effector protein (138–141). Caspase-1 cleaves pro-interleukins (IL-1β, IL-18, and IL-23), which results in sterile inflammation and lytic hepatocyte cell death (i.e., pyroptosis). Activation of the NLRP3 inflammasome has been found in murine models of both NAFLD and ALD (142–144). Also in rodent models of NAFLD/NASH, mRNAs encoding the NLRP3 inflammasome complex are elevated and overexpression of NLRP3 is associated with greater degrees of liver injury (145–147). Despite the similarities, the cell types involved and the trigger signals for NLRP3 activation appear to be somewhat different between ALD and NAFLD (148). In ALD, inflammasome and IL-1 production is increased at very early stages of the disease, which seems not to occur early in NASH (147). Also, in ALD, inflammasome activation is predominantly seen in Kupffer cells, while in NASH NLRP3 is mainly activated by hepatocytes (149–151). Finally, inflammasome component deficiency, in mouse models, protects against inflammation, steatosis, and liver injury in both ALD and NASH (152–154).

### Extracellular Vesicles and MicroRNAs

In response to injury, damaged cells release extracellular vesicles (EVs) which are membrane-surrounded structures (66) released by almost all types of cells. EVs can contain a wide variety of cargoes [e.g., proteins, lipids, and nucleic acids [coding and non-coding RNA] and mitochondrial DNA] that mediate intercellular communication. In the setting of liver damage, hepatocytes increase EV release, which may act on different target cells leading to pivotal pathobiological processes, such as activation of macrophages, endothelial cells, and HSCs, thus promoting proinflammatory, angiogenic, and fibrotic responses (155–158). Observations made in mouse models suggest that these EV-mediated processes are relevant events in the pathogenesis of both NAFLD and ALD (159–161). Moreover, EVs are promising candidates to serve as disease biomarkers. Also, their therapeutic use as a liver-specific delivery method of different compounds is being studied (155). For an in-depth discussion of current knowledge about the role of EVs in NAFLD and ALD, the reader is referred to a recent review (155).

Changes in microRNA (miR) expression are involved in pathogenesis ALD and NAFLD. In NASH, it has been shown that an upregulation of miR-34a and a downregulation of let7d (miR precursor) decrease FAO, promoting fat synthesis in murine models (83, 162, 163), while miR-122 has been linked with steatosis and fibrosis (164). On the other hand, miR-132 may trigger fibrogenesis secondary to ethanol intake, and miR-155 is associated with ethanol-induced inflammation, probably mediated by TNF-α (165). In mouse models of NASH, miR-155 is also induced without a defined role (166, 167).

### Microbiota

Another interesting factor in the pathogenesis of ALD and NAFLD is the effect of the intestinal microbiota. The number of microorganisms inhabiting the gastrointestinal tract has been estimated to exceed 10^14^, with extremely diverse features (168, 169). Dysbiosis has been described in both ALD and NAFLD; however, the exact role in the NAFLD and ALD disease processes remains unclear (170). The intestinal microbiota composition is associated with the stage of fibrosis and also on the ethanol consumption pattern, being different between chronic, binge, and “social” drinkers (132, 171, 172). Chronic alcohol consumption disrupts tight-junction proteins and increases intestinal permeability, resulting in increased translocation of endotoxins (lipopolysaccharides) and bacterial DNA into the portal circulation, which increases even more by the overgrowth of gram-negative bacteria. This process activates Kupffer cells through activation of TLRs (TLR4 and TLR9) (173, 174), which may also contribute to steatosis and hepatic fibrosis via stimulation of TLR9-dependent profibrotic pathways in mouse models (152, 175). The peptidoglycan and flagellin, other bacteria-derived toxins, also have an impact on TLR signaling producing proinflammatory cytokines (176). Changes in microbiota have been described in NAFLD (mainly decreased *Bacteroidetes* and increased *Prevotella* and *Porphyromonas* species) (177). This dysbiosis may be an important factor in causing NASH, in mouse models and human, through different mechanisms like deregulating energy homeostasis, modulation of choline and bile acid metabolism, and generation of bacteria-derived toxins, such as lipopolysaccharide (LPS), and increased hepatic TNF-a expression (through TLR4 and TLR9-dependent profibrotic pathways) in hepatic Kupffer cells (178–181). Therefore, microbiota plays an important role in ALD and NAFLD, but with some differences. The TLR4 can activate two distinct pathways: one pathway is MyD88-dependent (producing activation of NF-κB and proinflammatory cytokines), and the other pathway is MyD88-independent (inducing type I IFNs and NF-κB) (151). MyD88-dependent signaling seems to have a relevant role in NAFLD, but not in ALD (*in vitro* and *in vivo* in murine models) (152, 182, 183). Furthermore, a role may have the adipocytokines that can inhibit MyD88-dependent pathways in macrophages (184).

### Bile Acids and Nuclear Receptors

Nuclear receptors (NR) are ligand-activated transcription factors that have a key role in regulating lipid homeostasis and inflammation in the NAFLD/NASH process. The NRs act as receptors for fatty acids, cholesterol, oxysterols, and xenobiotics and regulate the cell metabolism, cell differentiation, and cellular homeostasis. The principal NR studied in NAFLD/NASH are liver X receptor (LXR), pregnane X receptor (PXR), peroxisome proliferator-activated receptor-gamma (PPARγ), Farnesoid X receptor (FXR), and HNF4α, hepatocyte nuclear factor 4α (185–189). PPARα has been studied in NAFLD and ALD. PPARα induces FAO in the mitochondria, thus decreasing steatosis. PPARγ ligands can inhibit inflammatory responses by decreasing IL-6, TNF-α, and IL-1β secretion and iNOS production in macrophages and Kupffer cells (187, 188, 190). Polymorphism in the PPARγ gene is associated with the susceptibility to NAFLD. LXR and RXRα also play a role in ALD due to their actions in lipid homeostasis and inflammation, with a particular role of RXRα in alcohol detoxification. HNF4α is constitutively active through the binding of integral fatty acids. HNF4α has an important role in the maintenance of hepatocyte differentiation and the regulation of bile acid and lipid homeostasis genes, mainly in mouse models (191–193).

Bile acids (BA) are not only detergents that stimulate hepatic bile flow and biliary excretion and aid in the digestion and absorption of fats from the intestinal lumen but also relevant signaling molecules that act on hepatic and extrahepatic tissues to regulate lipid and carbohydrate metabolic pathways (77). FXR is highly expressed in the liver, small intestinal mucosa, and kidneys, with effects on glucose and lipid metabolism; acts as a sensor for BA; and regulates the BA synthesis, protecting hepatocytes from the toxic effect of BA and reducing the triglyceride levels (194). FXR has anti-inflammatory and anti-steatotic effects, promoting FAO through upregulation of PPARα and repressing lipogenesis (by the modulation of SREBP-1c expression). Activation of FXR in the ileal enterocytes after active intestinal BA uptake also has important metabolic implications via FXR-stimulated local production of FGF15 (FGF19 in humans) (195). In hepatocytes, FGF15/19 is a major regulator of BA synthesis, through FGF receptor 4 (FGFR4) (196), and also decreases hepatic lipogenesis and indirectly stimulates mitochondrial FAO, in mouse models (196, 197). FXR also has a beneficial role in glucose metabolism, and it is important in vascular remodeling (198–200). Experimental models with FXR-null mice fed a high-cholesterol/high-fat diet develop massive steatosis (201) and exhibit decreased insulin sensitivity. Conversely, treatment with the selective, non-steroidal FXR agonist GW4064 improved IR and glucose homeostasis in obese *ob/ob* and diabetic *db/db* mice (202). Many other specific BA-activated receptors, including members of the nuclear receptor superfamily (FXR, NR1H4), a vitamin D receptor (NR1I1), PXR (NR1I2), members of the G protein–coupled receptor superfamily (TGR5 and sphingosine 1 receptor 2), and transporters such as ileal apical sodium-dependent bile acid transporter (ASBT), have a role in insulin sensitivity and NAFLD pathogenesis and are a target for novel therapies.

## Conclusions

NAFLD and ALD share a number of features and often coexist. Alcohol consumption is often a confounding factor in patients with NAFLD due to inaccurate reporting of the magnitude of alcohol intake and the ill-defined impact of alcohol consumption, even within the arbitrary thresholds considered to diagnose NAFLD, on liver disease progression in these patients. Although initially some studies suggested protective effects in moderate doses, current evidence shows that there is no safe threshold for alcohol consumption in the setting of NAFLD. On the other hand, the presence of MetS and obesity increases the progression of ALD as well the incidence of HCC and mortality. Considering the high prevalence of obesity and MetS and the changing patterns of alcohol consumption worldwide, which may impact the incidence of advanced liver disease, it is necessary to better define both diseases, acknowledge the presence of a dual etiology of liver disease in a group of patients, and develop a multidisciplinary approach focused on preventive measures.

## Author Contributions

FI, AK, and OM contributed to the review concept and drafting of the manuscript. MA and JA contributed to the review concept, critical revision of the manuscript for important intellectual content, supervision, and final version approval. All authors contributed to the article and approved the submitted version.

## Conflict of Interest

The authors declare that the research was conducted in the absence of any commercial or financial relationships that could be construed as a potential conflict of interest.

## Abbreviations

NAFLD: non-alcoholic fatty liver disease
ALD: alcohol-related liver disease
FFA: free fatty acids
IR: insulin resistance
ER: endoplasmic reticulum
ROS: reactive oxygen species
EV: extracellular vesicles
DAMPs: damage-associated molecular patterns
HCC: hepatocellular carcinoma
HSC: hepatic stellate cell
IL-6: interleukin 6
LPS: lipopolysaccharide
MetS: metabolic syndrome
PDGF: platelet-derived growth factor
TLR: toll-like receptors
TLR4: Toll-like receptor 4
TNFα: tumor necrosis factor α
TGF-β: transforming growth factor-beta
T2DM: type 2 diabetes mellitus
DNL: *de novo* hepatic lipogenesis
FAO: fatty acid oxidation
ACC: acetyl-CoA carboxylase
CPT: carnitine palmitoyltransferase
IRS: insulin receptor substrate
TRAIL-R2: TRAIL receptor 2
HMGB1: high-mobility-group protein box
SHH: sonic hedgehog
NE: neutrophil elastase
NETs: neutrophil extracellular traps
NLRs: NOD-like receptors
miRs: microRNAs
NR: nuclear receptors
LXR: liver X receptor
PXR: pregnane X receptor
PPARγ: peroxisome proliferator-activated receptor-gamma
FXR: farnesoid X receptor
FGFR4: FGF receptor 4
ASBT: apical sodium-dependent bile acid transporter
BMI: body mass index
ALT: serum alanine aminotransferase
MAFLD: metabolic (dysfunction) associated fatty liver disease
AAFLD: alcohol-associated fatty liver disease.
